# A highly stable, nanotube-enhanced, CMOS-MEMS thermal emitter for mid-IR gas sensing

**DOI:** 10.1038/s41598-021-02121-5

**Published:** 2021-11-25

**Authors:** Daniel Popa, Richard Hopper, Syed Zeeshan Ali, Matthew Thomas Cole, Ye Fan, Vlad-Petru Veigang-Radulescu, Rohit Chikkaraddy, Jayakrupakar Nallala, Yuxin Xing, Jack Alexander-Webber, Stephan Hofmann, Andrea De Luca, Julian William Gardner, Florin Udrea

**Affiliations:** 1 Department of Engineering, University of Cambridge, Cambridge, CB3 0FA UK; 2 Flusso Limited, Cambridge, CB4 0DL UK; 3 Department of Electronic and Electrical Engineering, University of Bath, Bath, BA2 7AY UK; 4 Department of Physics, University of Cambridge, Cambridge, CB3 0HE UK; 5 Biomedical Physics, School of Physics and Astronomy, University of Exeter, Exeter, EX4 4QL UK; 6 School of Engineering, University of Warwick, Coventry, CV4 7AL UK

**Keywords:** Environmental impact, Quality of life, Electrical and electronic engineering, Sensors and biosensors, Carbon nanotubes and fullerenes, Infrared spectroscopy

## Abstract

The gas sensor market is growing fast, driven by many socioeconomic and industrial factors. Mid-infrared (MIR) gas sensors offer excellent performance for an increasing number of sensing applications in healthcare, smart homes, and the automotive sector. Having access to low-cost, miniaturized, energy efficient light sources is of critical importance for the monolithic integration of MIR sensors. Here, we present an on-chip broadband thermal MIR source fabricated by combining a complementary metal oxide semiconductor (CMOS) micro-hotplate with a dielectric-encapsulated carbon nanotube (CNT) blackbody layer. The micro-hotplate was used during fabrication as a micro-reactor to facilitate high temperature (>700 $^{\circ }$C) growth of the CNT layer and also for post-growth thermal annealing. We demonstrate, for the first time, stable extended operation in air of devices with a dielectric-encapsulated CNT layer at heater temperatures above 600 $^{\circ }$C. The demonstrated devices exhibit almost unitary emissivity across the entire MIR spectrum, offering an ideal solution for low-cost, highly-integrated MIR spectroscopy for the Internet of Things.

## Introduction

Gas sensors are at the center of increasing research and development efforts, driven by many scientific, industrial and commercial applications^1^. These include the monitoring of environmental pollutants from deforestation^2^, vehicles and industry^3^ and also air quality within buildings^4^. There is an increased awareness of the impact of air pollution on human health^3^, leading to a rise in demand for low cost, accessible, compact, and readily deployable air quality monitoring^5^. To sustain the emerging global demand, gas sensors must meet a suitable and challenging balance between performance and cost^1^. In addition to being economically viable, an increasing number of sensors have stringent power and volume constraints^1^, e.g. those deployed within the Internet of Things (IoT)^6^ and in mobile platforms^7^. These requirements motivate researchers to explore novel materials, designs and technologies to achieve miniaturization, monolithic integration of components, low cost, reduced power consumption and manufacturability^1^.

Amongst the various different sensing technologies, optical gas sensors offer several advantages in terms of selectivity and long-term operational stability^1^. Notably, nondispersive infrared (NDIR) sensors currently dominate the carbon dioxide (CO$_2$) gas sensor market, and also serve many other applications^8^. However, despite their inherent advantages (e.g., for spectroscopic sensing), NDIR gas sensors are currently mostly employed for the detection of single analytes, or a few species at the same time. A limit to wider adoption has been availability of miniaturised broadband MIR light sources which are low cost and optically efficient (arguably the core of an optical gas sensor)^1^. Bulb based thermal sources have traditionally been used but are fragile, bulky and have limited optical efficiency at wavelengths above 5 $\upmu$m. Light-emitting diodes (LED) offer improved integration and reliability but are costlier to fabricate due to the use of specialist III–V semiconductor technologies^9^.

Exploiting standard complementary metal-oxide semiconductor (CMOS) processes is an attractive way to fabricate low-cost integrated thermal MIR sources and detectors, and has led to many innovative micro-electro-mechanical system (MEMS) based devices^1,10^. Various techniques have been proposed to enhance the emissivity/absorptivity^1^ of CMOS-MEMS thermal devices, including the use of carbon nanotubes (CNTs) adlayers offering near unit broadband emissivity^11,12^, or plasmonic metamaterials for specific MIR bands^13,14^. Multi-species spectroscopic detection requires the MIR source to operate at an ensemble of target MIR wavebands, making the overall CNT broadband emission enhancement^11,12^ attractive for spectroscopy^1^. However, despite their blackbody-like advantages^15,16^, thus far most research has observed such CNT, and in general all graphitic nanocarbon adlayers, burning off in air when operated at temperatures above 400 $^{\circ }$C^17,18^. This poses a limit (optical emission and operational stability) to their integration into CMOS MEMS micro-hotplate MIR sources, which are typically operated at these temperatures^19^. Although the use of an inert gas can be used to prevent CNT burn-off, this requires the use of specialist hermetically sealed ceramic or metal packages, which can significantly impact cost^20,21^.

Here, we present a solid-state approach based on a dielectric-encapsulation method that enables the long-term operational stability of CNT-coated thermal emitters. We show that alumina (Al$_2$O$_3$)-encapsulated CNTs grown on a MEMS micro-hotplate can withstand temperatures in excess of 800 $^{\circ }$C when operated in air. The encapsulated CNT adlayer has an emissivity close to unity (a $\sim$ 8-fold increase with a reference to a standard MEMS device) and demonstrates stable operation at 600 $^{\circ }$C for 10 days. The work paves the way for encapsulation techniques to be more widely applied to temperature and air sensitive nanomaterials, allowing them to operate stably in air, well above their normal temperature threshold.

## Results

**Figure 1:**
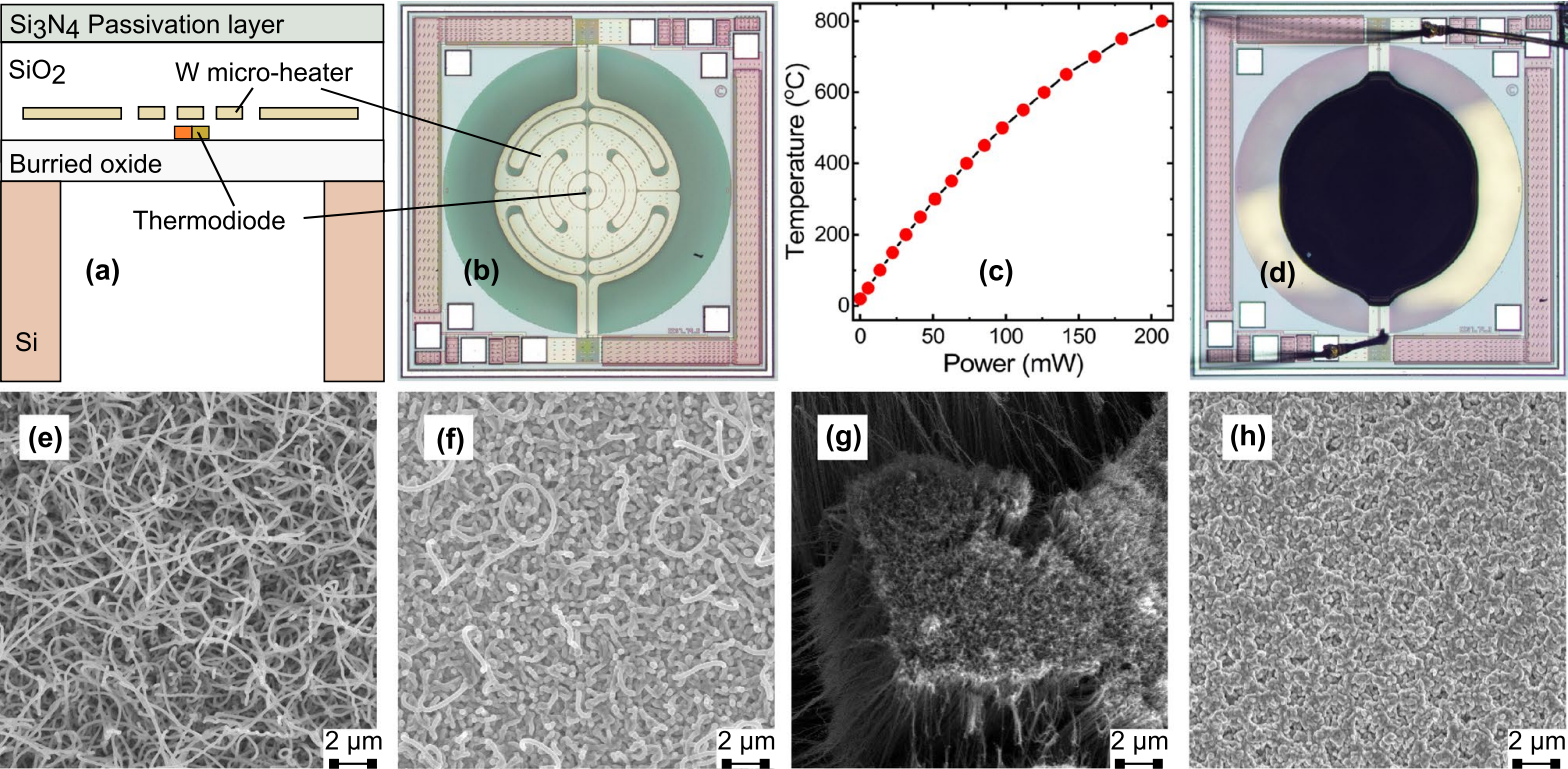
Device fabrication. (**a**) Micro-hotplate cross-section (not to scale) employing a tungsten (W) heating element embedded into a $\sim$ 5 $\upmu$m thick silicon dioxide (SiO$_2$) membrane formed by deep reactive ion etching. (**b**) Optical image of the micro-hotplate, showing a multi-ring designed heating element surrounded by the membrane. Chip size = 1.76 mm $\times$ 1.76 mm. (**c**) Micro-heater temperature as a function of power consumption. (**d**) The active heating element serves three purposes: (i) localised in-situ heating (micro-reactor) during the CNT growth process; (ii) employed as thermal profiler (adlayer flashing) for self-annealing; and (iii) used to generate IR emission during device operation (typically at temperatures of 300–600 $^{\circ }$C). (**e**) Typical SEM image of spaghetti-like CNT structure grown by an in-situ CVD process using acetylene (C$_2$H$_2$) blended with ammonia (NH$_3$) over an iron (Fe) catalyst, and (**f**) their SEM image after $\sim$ 50 nm alumina (Al$_2$O$_3$) encapsulation. (**e**) SEM image of denser, more aligned CNTs synthesised by mixing C$_2$H$_2$ with hydrogen (H$_2$), and (**h**) their SEM image after encapsulation.

For our experiment, we use an in-house designed micro-hotplate fabricated at a commercial foundry. The micro-hotplate cross-section is shown in Fig. 1a and consists of a multi-ring resistive tungsten (W) heating element (800 $\upmu$m diameter) embedded within a $\sim$5 $\upmu$m thick silicon dioxide (SiO$_2$) membrane (1200 $\upmu$m diameter), to ensure low direct current (DC) power consumption^19^. W was chosen as the heating element and interconnection metal due to its superior resistance toward electromigration and higher glass transition temperature when compared to doped poly-silicon or aluminum^10,19^. The micro-hotplate is built in a CMOS-SOI (silicon on insulator) technology and features a monolithically integrated, monocrystalline silicon thermodiode which can operate linearly with high accuracy at record temperatures up to 600 $^{\circ }$C^22^. The thermodiode can be used as an accurate temperature sensor when the micro-hotplate is used as an IR emitter and when calibrated allows resolutions below 0.5 $^{\circ }$C. For the growth of the CNTs and operation above 600 $^{\circ }$C it is however preferred to use the W heater as a resistive temperature detector (RTD). W has a large and a stable temperature coefficient of resistance (TCR) ($\sim$ 4.5 $\times$ 10$^{-3}$ K$^{-1}$) and has been shown to function up to 1000 $^{\circ }$C with relatively high accuracy $\sim$ 2 $^{\circ }$C^19^. Micro-hotplates can reach temperatures in excess of 700 $^{\circ }$C and have fast thermal transient times > 4 $\times$ 10$^4$ $^{\circ }$C/s, enabling voltage-controlled thermal ramps and stable MIR emission with excellent reproducibility at very low cost^10,19^. An optical image of our fabricated micro-hotplate, showing the heating element surrounded by the membrane is given in Fig. 1b.

We use an in-situ chemical vapour deposition (CVD) process to integrate the CNT adlayer^11,12^. CVD processes typically require substrate temperatures in excess of 400 $^{\circ }$C^23,24^, which are not CMOS compatible, as temperature effects can damage the integrated circuitry (e.g. due to accelerated alloying and atomic migration)^10^. Due to the thermal isolation offered by the thin dielectric membrane, our micro-hotplates can easily reach temperatures in excess of 750 $^{\circ }$C (Fig. 1c) in localized “hot zones”, without compromising the functionalities of the peripheral CMOS circuitry placed on the chip substrate that are only a few micrometers distant from the hot zone. Hence, our micro-hotplates can be effectively employed as ideal CMOS compatible micro-reactors, allowing viable CNT-CMOS integration at wafer level^12^. In addition, our design allows for relatively low DC power consumption to achieve such high temperatures (e.g. $\sim$ 100 mW at 500 $^{\circ }$C, as shown in Fig. 1c), which can be further minimized by using modulated driving signals (e.g. 50% duty cycle in our case). To test the encapsulation efficiency, we used two commonly used process gases, ammonia (NH$_3$)^12^ and hydrogen (H$_2$)^11^, blended with the same carbon-containing gas [acetylene (C$_2$H$_2$)], over an iron (Fe) catalyst process. As reported elsewhere in the CNT-synthesis literature, using NH$_3$ tends to give more spaghetti-like bundles of nanotubes^12^, whilst using H$_2$ gives more spatially dense, vertically aligned nanotubes^11^, thus providing us with a comprehensive testbed (impacts of CNT crystallography and morphology) for our experiment. An optical image for a NH$_3$:C$_2$H$_2$-synthesised CNT sample is shown in Fig. 1d. Scanning electron microscopy (SEM) inspection confirms the successful growth of both spaghetti-like (Fig. 1e) and vertically aligned (Fig. 1g) nanotube forests.

**Figure 2:**
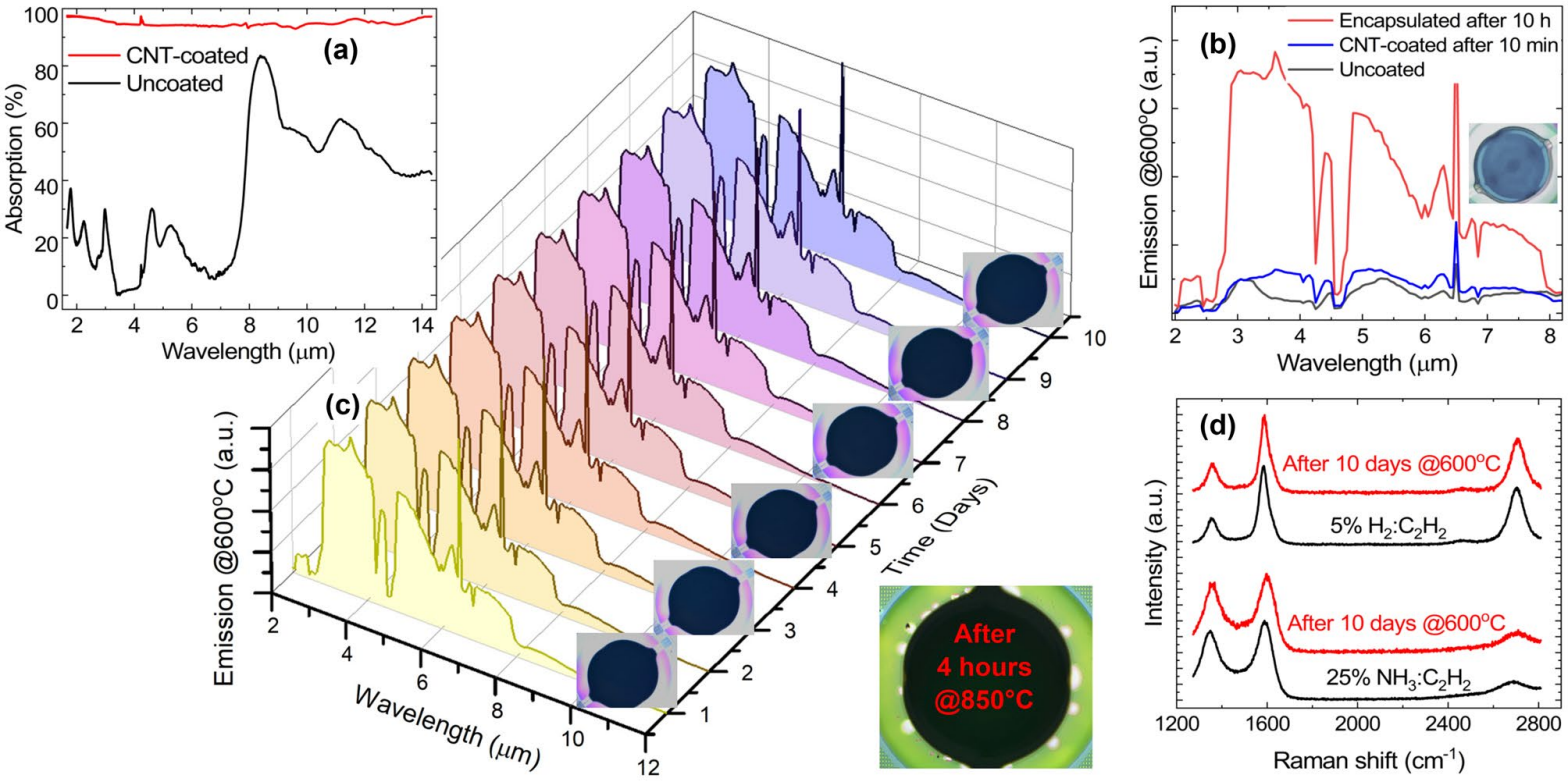
Characterization and stability tests in air. (**a**) Infrared absorption spectra of an uncoated micro-hotplate (black line) compared to that of a CNT-coated plate (red line) absorbing almost all light. (**b**) Emission spectra measured at 600 $^{\circ }$C for an uncoated (black line), CNT-coated (blue line), and encapsulated (red line) micro-hotplate, respectively. CNT-coated devices show a $\sim$ 8-fold emission increase when compared to uncoated devices at equivalent temperature, however, unencapsulated CNTs burn-off within minutes, resulting in their emission spectrum (blue line) falling from an initial value close to that of an encapsulated device (red line) to that of an uncoated device (black line). Optical inspection (inset) shows CNTs have almost entirely burned-off. (**c**) Emission spectra measured at 600 $^{\circ }$C over a 10-day period, for a device with encapsulated NH$_3$:C$_2$H$_2$-synthesised CNTs (shown in Fig. 1f). Optical images recorded at respective times show encapsulated CNTs are not affected by the high temperature operation. An optical image of a test sample after operating at a much higher, 850 $^{\circ }$C, temperature for 4 hours is presented in the bottom-right corner, showing the CNTs are intact. (**d**) (bottom two lines) Raman spectra measured at 532 nm before and after the operation stability test presented in (**c**). As a comparison, the top two lines show the Raman spectra measured for the same test (not shown) done with H$_2$:C$_2$H$_2$-synthesised CNTs (shown in Fig. 1h) instead, showing both samples remain stable.

It is known that CNTs burn in air (typically within minutes) at temperatures above 400 $^{\circ }$C, depending on their diameter, number of walls or amount of defects^17,18^, thus hindering their application as thermal emitters. To isolate the air-exposed CNTs from oxidation^17,18^, and create a thermally stable emitter interface, we encapsulate the as-grown CNTs in a $\sim$ 50 nm thick atomic layer deposited (ALD) Al$_2$O$_3$ coating. Al$_2$O$_3$-based encapsulation guarantees high thermo-mechanical stability, and was shown to have the capability of operation at temperatures above 500 $^{\circ }$C, while proving a good barrier for oxygen (O$_2$) and water (H$_2$O)^25,26^. Figure 1f,h show the SEM images, after encapsulation, of the CNTs shown in Fig. 1e (spaghetti-like NH$_3$:C$_2$H$_2$-synthesised) and g (aligned H$_2$:C$_2$H$_2$-synthesised) respectively. In order to improve the thermal stability after encapsulation, we used a device self-annealing^26,27^ process enabled, in our case, by the micro-heaters themselves. Thermal profiles were applied with electro-thermal modulation in $\sim$ 100 $^{\circ }$C steps with temperatures of up to $\sim$ 800 $^{\circ }$C; this aligned the induced thermo-mechanical stress on the membrane with the operating frequency thereby avoiding membrane breakages.

A typical optical absorption spectrum (see “Methods”) for our micro-hotplates, in the 2–14 $\upmu$m waveband, is shown in Fig. 2a (black line), with an absorption peak of $\sim$ 85% at 8.5 $\upmu$m; a signature of the Si–O stretching vibrations within the SiO$_2$ membrane^28,29^. In the same figure (red line), a micro-hotplate with an in-situ grown NH$_3$:C$_2$H$_2$-synthesised CNT (Fig. 1f) layer exhibits almost 100% absorption, a behaviour attributed to the CNT layer’s blackbody-like nature^15,16^. To study the emission properties of our uncoated, CNT-coated and encapsulated devices, we implemented a proportional integral derivative (PID)-based temperature controller, able to control the self-heating micro-hotplate temperature within $\sim$ 0.5 $^{\circ }$C resolution. The emission spectra of the self-heated devices was then measured by a MIR spectrometer (Bentham) (Fig. 2b). A representative spectrum, recorded at 600 $^{\circ }$C, for an encapsulated (NH$_3$:C$_2$H$_2$) sample, is shown in Fig. 2b (red line), showing a $\sim$ 8-fold emission enhancement when compared to an uncoated device operated at the same temperature (black line). We also measured the emission spectra of unencapsulated CNT-coated devices which, as expected, exhibited a rapid ($\sim$ minutes) decrease in emission at elevated temperature, consistent with CNTs burning off when exposed to air^17^. An example spectrum is shown in Fig. 2b (blue line), recorded after $\sim$ 10 min of operation at 600 $^{\circ }$C. A visual comparison between black and blue lines suggests that most CNTs had burnt off, confirmed by a subsequent visual inspection under a microscope (Fig. 2b, inset).

To investigate the long-term operational stability of our encapsulated devices, we performed stress testing in air under normal room conditions [standard pressure (1010–1020 mbar), temperature (18–21 $^{\circ }$C) and relative humidity (30–50%)] at temperatures up to 900 $^{\circ }$C, which are significantly higher than the actual device operating temperature ($\sim$ 500–600 $^{\circ }$C). Typical emission spectra, for a device operated continuously at 600 $^{\circ }$C over a 10 day period (recorded periodically every 24 h), are presented in Fig. 2c, with $\sim$ 3 $\times$ 10$^{-5}$ standard deviation variation, indicating excellent stability. Optical images, recorded at representative times during the test, are also presented, which show that the encapsulated CNTs are physically unaffected by high operating temperature. Devices with encapsulated in-situ grown CNTs (synthesised in both H$_2$ or NH$_3$) were found to have stable and reproducible emission spectra when operated for hours at temperatures of up to 900 $^{\circ }$C. An optical image of a sample is shown in the bottom-right corner of Fig. 2c, recorded after 4 h of operation in air at 850 $^{\circ }$C, further showing the physical stability of the CNTs at high temperature. It is worth noting that at such high temperatures some membranes (not the CNTs) failed, due to extreme thermally-induced mechanical stress. Failed devices were analysed by optical and SEM imaging and the failures identified were down to membrane breakage, rather than burn-off of the encapsulated CNT layer which stayed intact, even at the extreme temperatures. Similar membrane breakage at extreme temperatures has been observed with non-coated devices (without the CNT layer). To check the quality of the encapsulated CNTs before and after the 10-day operation test, we characterized them using Raman spectroscopy. Figure 2d (bottom two lines) plots the Raman spectra of the devices presented above. We do not observe any change in the D peak position, width and intensity, indicating the long-term high-temperature operation does not induce additional defects with respect to the starting material^30^. As a comparison (see top two lines), we also present the Raman spectra for the same test ran with H$_2$:C$_2$H$_2$ encapsulated CNTs instead, showing good operational stability for both samples, thus highlighting the broader application potential of our encapsulation technique.

**Figure 3:**
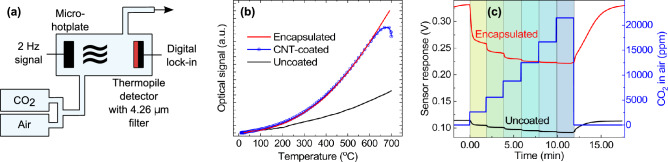
Nondispersive infrared (NDIR) experiment. (**a**) Schematic of the NDIR gas sensor setup. (**b**) Relative optical response, recorded by the thermopile at 4.26 $\upmu$m, for an uncoated (black line), CNT-coated (dotted blue line), and encapsulated (red line) micro-hotplate respectively. The detected IR emission from the unencapsulated device (dotted blue line) drops rapidly (minutes) at temperatures above 600 $^{\circ }$C, consistent with data presented in Fig. 2b. (**c**) CO$_2$ sensor response voltage with an uncoated (black line), and encapsulated (red line) micro-hotplate respectively. A $\sim$ 8-fold increase in relative voltage $\Delta V=V_{0 ppm}-V_{lock-in}$ can be observed for the encapsulated CNT device.

To test the performance of our MIR emitter in an application type setup, samples were benchmarked against an uncoated device using a custom made NDIR gas sensor designed for CO$_2$ detection. A schematic of our experimental setup is shown in Fig. 3a. We use our thermal emitter as a MIR light source, coupled to a single-channel thermopile detector (Heimann HMS-J21) with integrated 4.26 $\upmu$m filter tuned to the CO$_2$ absorption waveband. Both emitter and detector are fitted in a custom gas cell with an optical path length of 4 cm between emitter and detector. The gas cell is connected to a gas mixing system, enabling the CO$_2$ concentration ($c_{CO_2}$) to be controlled by mixing $c_{CO_2}$ at 5$\%$ from a cyclinder with dry air. Our custom-built experimental setup allows CO$_2$ concentrations to be controlled below 100 ppm with a minimum controlled change in concentration of a few ppm. The relative optical signal recorded by the thermopile at room conditions (see “Methods”) is shown in Fig. 3b. A CNT (NH$_3$:C$_2$H$_2$-synthesised)-coated device (dotted blue line) gives a similar response to that of an encapsulated device (red line) to temperatures up to $\sim$ 600 $^{\circ }$C, but the emission level starts to decrease rapidly (within minutes) towards that of a typical uncoated device, confirming the results presented in Fig. 2b. We found no noticeable change in the response of the device encapsulating CNTs (Fig. 3b, red line) after several weeks of operation. Figure 3c shows the sensor’s voltage response with an encapsulated CNT device (red line), compared to an uncoated device (black line) with $c_{CO_2}$ ranging from 0 to $\sim$ 21,500 ppm. The encapsulated CNT device gives a response of $\Delta V=V_{0 ppm}-V_{lock-in}$ = 111 mV at maximum CO$_2$ concentration, compared to only 22 mV for the uncoated device. The standard deviation of the CO$_2$ measurements is $\sim$0.6 mV, which is much less than the amplitude recovered signal post amplification 200–300 mV, with the signal-to-noise ratio (SNR) mainly limited by thermal noise. Considering a relative sensitivity defined as $S=\frac{d\Delta V}{dc_{CO_2}}|_{c_{CO_2}=0}$ and a $\sim$ 3 dB measured SNR, we estimate a $\sim$ 0.12 ppm limit of detection for an encapsulated device compared to $\sim$ 1 ppm for an uncoated one, consistent with the results in Fig. 2b.

## Discussion

In conclusion, we demonstrate an efficient CMOS-compatible CNT-encapsulated micro-hotplate-based MIR emitter fabricated on a single SiO$_2$ dielectric membrane, with near unity emissivity, and long-term operational stability. Processing of the chip is simplified by employing standard CMOS tungsten heaters as micro-reactors for precise in-situ CNT growth, allowing scalable integration at waver level. The same heating elements also serve as enablers for versatile self-annealing thermal profiles that can be adapted for easy optimization of various processes. We show stable operation for alumina-encapsulated CNTs up to record temperatures of 900 $^{\circ }$C. Employing the emitter in a proof-of-concept optical sensing demonstration, we measured a 8-fold increase in relative sensitivity to CO$_2$ compared to the use of a conventional MEMS emitter. Our emitter exhibits almost unitary emissivity across the entire MIR band, making it particularly attractive for a variety of low-cost, low-power and high volume spectroscopic applications in the MIR spectral region.

## Methods

The micro-hotplates were designed in Cadence$\copyright$ and fabricated using a commercial 1 $\upmu$m SOI-CMOS process on 6 inch silicon (Si) wafers. The membrane was formed by deep reactive ion etching (DRIE) of a 400 $\upmu$m thick Si substrate, with the buried SiO$_2$ layer acting as an etch stop. A silicon nitride (Si$_3$N$_4$) passivation layer shields the membrane from environmental factors, such as humidity^31^.

CNTs were grown by an in-situ thermal CVD of C$_2$H$_2$ over a Fe catalyst process. The micro-hotplates were coated (ALD) with a $\sim$ 10 nm Al$_2$O$_3$ then sputtered with a 2–4 nm Fe catalyst. The devices [mounted onto TO-type packages connected to a power supply (Keithley 2400)], were then transferred to a custom-build CVD chamber, to grow the CNTs, which was pumped down to a base pressure of $\sim$ 0.5 mbar. The CNT growth process was optimized by a $\sim$ 0.5 $^{\circ }$C resolution PID-based temperature controller, implemented in LabVIEW© software, which was set to a 20 $^{\circ }$C/s heating rate. High purity NH$_3$ or H$_2$ was introduced into the chamber when the microheater reached $\sim$ 500 $^{\circ }$C, and then operated at 725 $^{\circ }$C for 60 s to form the small catalyst Fe islands. C$_2$H$_2$ was then introduced through a separate line in a 5% H$_2$:C$_2$H$_2$ or 25% NH$_3$:C$_2$H$_2$ atmosphere respectively, maintained to $\sim$ 4 mbar during a $\sim$ 10 min growth process. Devices were then introduced into an ALD reactor (Cambridge NanoTech) to deposit $\sim$ 50 nm Al$_2$O$_3$ [using trimethylaluminium (TMA) and water (H$_2$O) as precursors at 200 $^{\circ }$C] for encapsulation, followed by a self-annealing process at $\sim$ 400, 500, 600, and 700 $^{\circ }$C, for 30 min each, respectively.

To obtain the optical absorption (A) spectral profiles, in the wavelength range of 2–14 $\upmu$m, transmission (T) and reflection (R) FTIR measurements (normal incidence) were coupled to give A = 1-R-T. The optical aperture of the micro-FTIR system (Agilent Cary 620 FTIR Microscope) was set to image only the heater area of the micro-hotplate. The emission spectral profiles were measured by mounting the devices to a bespoke MIR spectrometer (Bentham) composed by a monochromator (TMc300) connected to a cryogenically-cooled mercury cadmium telluride detector (DH-MTC). Raman spectra were acquired by a Renishaw inVia Raman microscope at 532 nm excitation.

The NDIR sensor was interfaced to a National Instruments DAQ card (NI USB-6353) to allow for automatic control and data acquisition via LabVIEW© software. The microheater was voltage modulated by a 2 Hz periodic squarewave using a custom amplifier. A custom preamplifier (60 dB voltage gain) and software based lock-in amplifier (1 s integration time; $\sim$ 50 dB SNR) were used to recover the signal detected by the thermopile from the background noise. The full 16-bits of the A/D conversion were used for the measurement. The overall flow rate employed for the CO$_2$ sensing was 200 sccm, achieved using a combination of computer controlled mass flow controllers (MKS).
